# Crossover gait in running and measuring foot inversion angle at initial foot strike: a front-view video analysis approach

**DOI:** 10.3389/fbioe.2023.1210049

**Published:** 2023-07-14

**Authors:** Muhammad Nur Shahril Iskandar, Ray Ban Chuan Loh, Mee Yee Mavis Ho, Jing Wen Pan, Pui Wah Kong

**Affiliations:** ^1^ Physical Education and Sports Science Academic Group, National Institute of Education, Nanyang Technological University, Singapore, Singapore; ^2^ Sports Medicine and Surgery Clinic, Tan Tock Seng Hospital, Singapore, Singapore

**Keywords:** running-related injuries, 2D motion analysis, low-cost alternative, foot kinematics, gait analysis

## Abstract

**Introduction:** Foot inversion angle at initial foot strike is associated with various running-related injuries. Traditionally, video analysis of foot inversion angle has been accomplished by positioning a camera to record from the back view, but complications arise when a crossover gait obscures the area of measurement. This study aims to investigate the viability of measuring foot inversion angles at initial foot strike of running from the front view as an alternative to using the back view in 2D video analysis.

**Methods:** Forty-four healthy runners (20 females, 24 males) ran at their self-selected speeds on a treadmill with their gait recorded from front and back camera views. Foot inversion angles at initial foot strike were analyzed using Kinovea. A 2 × 2 (Camera × Foot) ANOVA with repeated measures was performed on the foot inversion angle data. Subsequently, correlation and linear regression were performed to determine the relationship between the back and front-view measurements.

**Results:** Thirteen runners (29.5%) displayed crossover gait within 18 gait cycles. ANOVA revealed a significant main effect on Camera (*p* < .001) only, where foot inversion angle was greater from the front camera view. Correlation analysis showed a significant positive correlation between the front and back camera views (*r* = 0.388, *p* < .001). Regression analyses yielded an equation, y = 0.42 + 0.53 x , where y and x were the foot inversion angle measured from the back and front camera views, respectively.

**Discussion:** With a linear regression conversion equation, front-view foot inversion angles at initial foot strike can be used to determine rearfoot inversion angles when crossover gait obstructs the back camera view.

## 1 Introduction

Running is a common form of physical activity that provides many health benefits and has attracted more and more participation in recent years (Vitti et al., 2020). However, running has been considered a high-risk physical activity due to the high incidence rate of overuse running-related injuries (RRIs) reported among novices (Kluitenberg et al., 2015; Kemler et al., 2018) and recreational runners (Hespanhol Junior et al., 2013). Novice runners are classified as individuals who have less than 1 year of regular running experience while recreational runners are classified as individuals who have been engaging in running activities minimally once per week for 1 year (Honert et al., 2020). Notably, a systematic review found that novice runners face a considerably greater risk of injury, with a rate of 17.8 injuries per 1,000 h of running, whereas recreational runners experienced 7.7 injuries per 1,000 h of running (Videbæk et al., 2015). In view of this, studies have sought to establish the biomechanical factors of various RRIs via gait analysis (Fatone & Stine, 2015; Ceyssens et al., 2019), to gain insights into the running techniques that may increase the injury risk. Thereafter, an appropriate rehabilitation or gait retraining program can be advised (Roper et al., 2016; Warne et al., 2017).

Gait analyses are commonly performed using either a two-dimensional (2D) video-based or three-dimensional (3D) motion capture system. Although 3D systems have been considered the gold standard, it requires expensive specialized equipment and would require some level of expertise to operate. Additionally, data collections are tedious and limited to laboratory settings. With these constraints, clinicians and researchers have explored the use of 2D video analysis (Damsted et al., 2015; Fatone et al., 2015; Hensley et al., 2021), which is relatively simple and of low cost (Fatone and Stine, 2015). Recorded videos can subsequently be integrated with commercially available video analysis software (e.g., Dartfish, Kinovea, and Hudl) that allow users to annotate at specific key points of interest (DeFroda et al., 2016) to obtain kinematic measurements. The portability of 2D video analysis also makes it a cost-effective tool for practitioners. In a recent study, Hensley et al. (2021) found that about 75% of practitioners use 2D video cameras during their clinical gait assessment. This highlights the ease of accessibility and popularity to conduct gait analysis using 2D video analysis among practitioners.

Previous studies have demonstrated moderate to high correlations between 2D video analysis and 3D motion capture when evaluating gait parameters in running (Maykut et al., 2015; Dingenen et al., 2018b; De Oliveira et al., 2019; Martinez et al., 2022). Specifically, Martinez et al. (2022) observed sagittal plane kinematic differences of 2.5°–5°, while Maykut et al. (2015) reported differences of 1°–4° for frontal plane kinematics. Multiple studies have also consistently reported excellent intra and inter-rater reliability in 2D analysis with an intra-class correlation coefficient exceeding 0.90, indicating strong reliability in 2D analysis (Maykut et al., 2015; Dingenen et al., 2018b; De Oliveria et al., 2019; Mousavi et al., 2020). Thus, these studies have shown compelling evidence that the application of gait analysis using 2D video analysis is possible.

**FIGURE 1:**
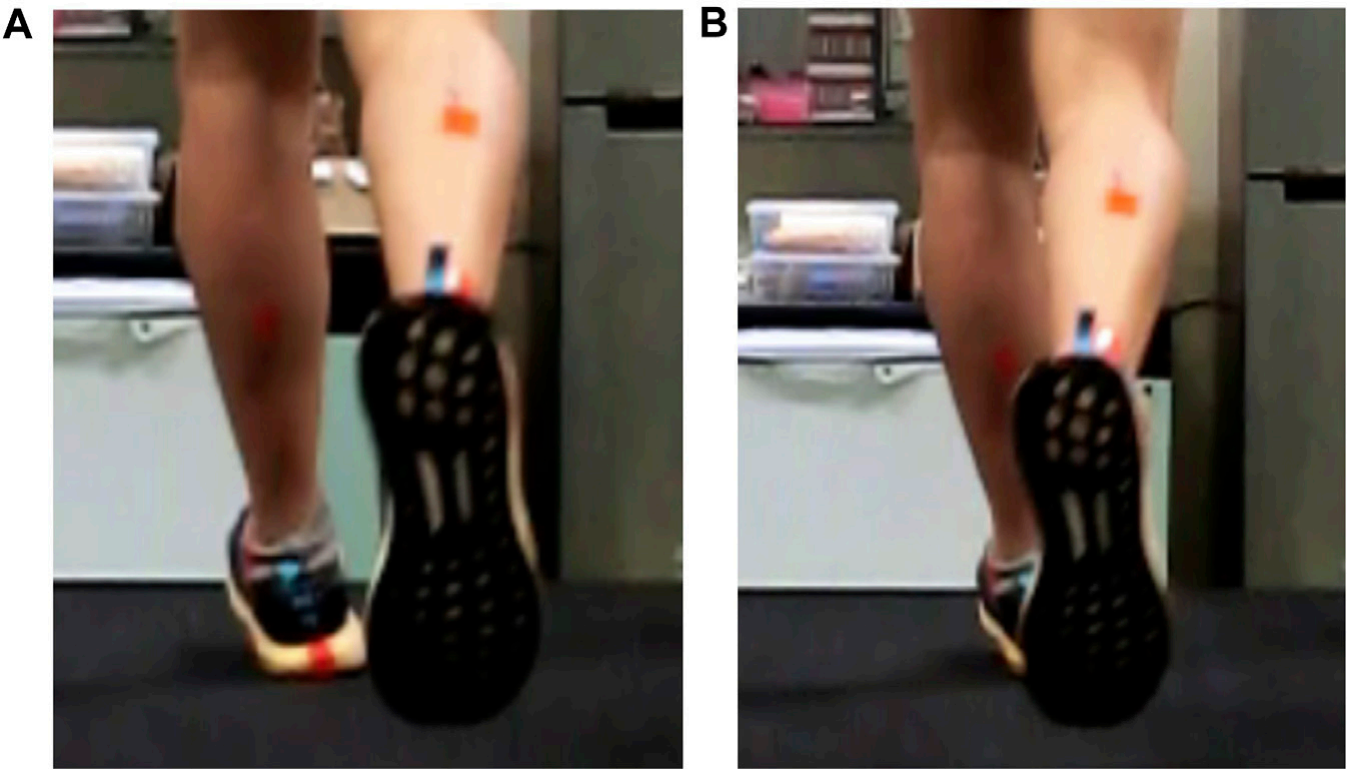
Example of foot inversion at initial foot strike captured from a back camera view. The initial foot strike from the back view is **(A)** unobstructed, and **(B)** blocked by the right foot.

Despite the aforementioned advantages of 2D video analysis, there are certain limitations when using it for gait analysis. One such limitation is ensuring the visibility of key landmarks at all times for post-processing analysis. In situations where occlusions of key landmarks occur, it may hinder clinicians and researchers to obtain measurements at a specific gait cycle event. In particular, the rearfoot inversion angle at the initial foot strike, typically measured from a back camera view (Cornwall and McPoil, 1995), is known to obstruct the visibility of key landmarks during post-processing analysis. The initial foot strike is the instance when the foot first makes contact with the ground which also marks the beginning and end of a gait cycle (Novacheck, 1998). A problem arises when runners display a crossover gait, where the planted foot is obstructed by the other foot in the swing phase (Figure 1). This phenomenon was also observed by Vincent et al. (2014). The rearfoot inversion angle at the initial foot strike holds significant importance as it is associated with RRIs such as iliotibial band syndrome (Grau et al., 2008) and Achilles tendinopathy (McCrory et al., 1999; Mousavi et al., 2019). Furthermore, the measurement of this angle is essential for evaluating other variables related to RRIs such as ankle eversion range of motion, eversion velocity, and eversion duration during the stance phase of running (Kuhman et al., 2016). However, the presence of a crossover gait in runners may hinder clinicians and researchers from obtaining accurate measurements of the foot inversion angle at the initial foot strike when recorded from the back camera view. Hence, an alternative camera view may help evaluate such gait variables.

Front-view video analysis poses a possible method to overcome the crossover gait problem since the obstruction only occurs in the dorsal plane. However, it remains unclear whether the measurements obtained from front and back views can be compared. The current study aimed to investigate the efficacy of using a front camera view in analyzing foot inversion angle during treadmill running. Based on a previous study that reported a positive correlation between static forefoot varus and rearfoot angle (Johanson et al., 2010), it was hypothesized that the foot inversion angle measured from front and back camera views would be comparable. The findings would be expected to provide valuable insights to clinicians and researchers in evaluating foot inversion angle among runners with crossover gait.

## 2 Materials and methods

### 2.1 Participants

Forty-four (20 females, 24 males) healthy recreational runners (age 25.2 ± 6.5 years; height 167.2 ± 7.0 cm; body mass 62.8 ± 8.1 kg) were recruited. The inclusion criteria for the study were: (1) individuals who were recreational runners, with more than 1 year of regular running months (Honert et al., 2020), (2) with a minimum of one weekly run for the past three, (3) between the ages of 18–45, (4) comfortable to run on a treadmill without the need for handrails, and (5) capable of running continuously at a speed of 8 km/h for at least 10 min (Gazendam & Hof, 2007). Participants were excluded from the study if they (1) were pregnant during the time of the study (for female participants), (2) answered positively to any questions on the Physical Activity Readiness Questionnaire (PARQ+ 2020) indicating the presence of serious health conditions, (3) had undergone surgery on the legs within the past year, or (4) had lower limbs injuries requiring more than 7 days of rest in the past 6 months. All participants provided written informed consent prior to their participation and ethics approval was granted by the Nanyang Technological University Institutional Review Board (IRB-2021-124).

### 2.2 Experimental setup

**FIGURE 2:**
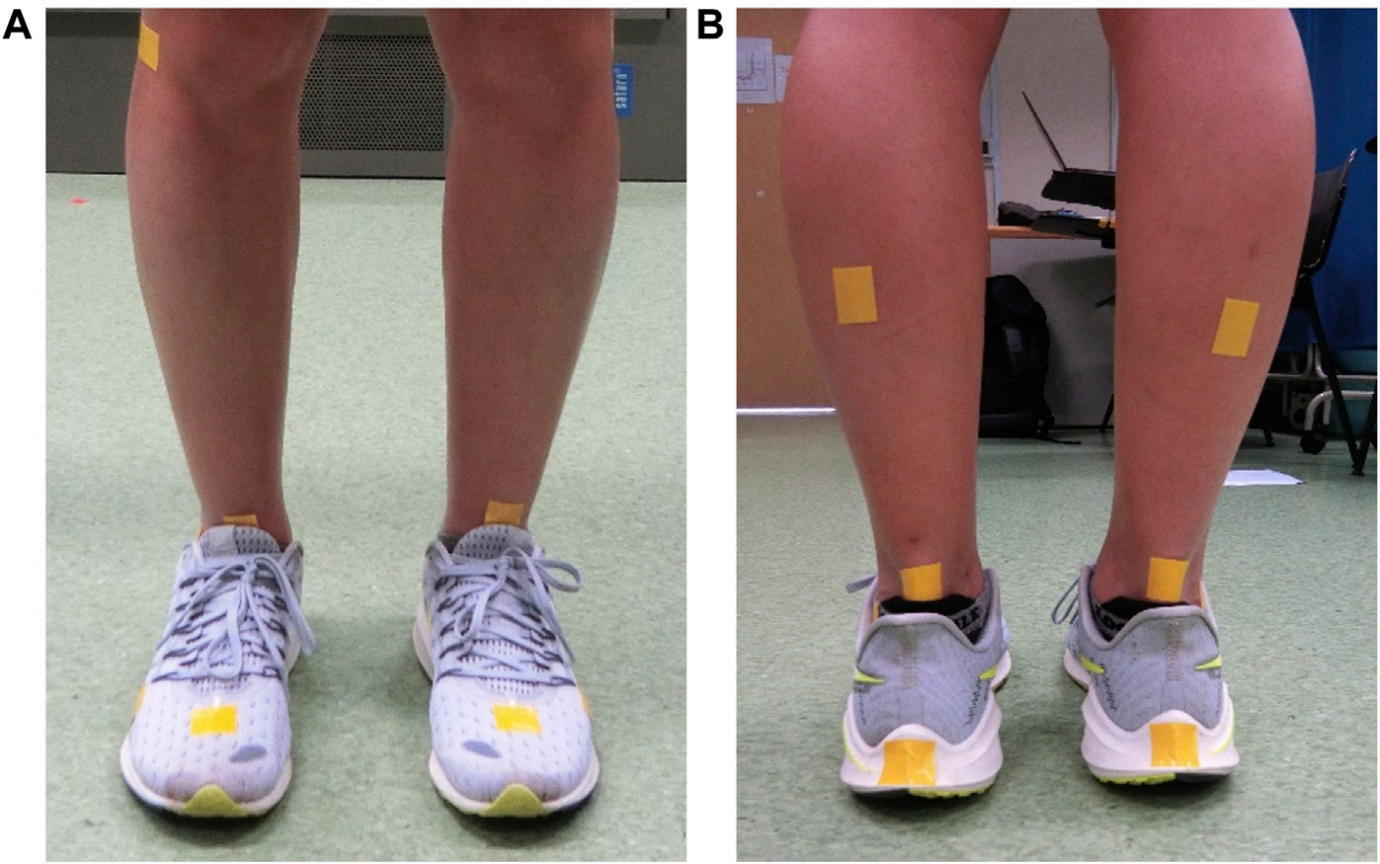
Marker placement using cloth tapes in **(A)** the front view and **(B)** the back view. Other markers not mentioned earlier are not relevant to this study.

All participants were invited to run at their comfortable paces on a treadmill (h/p/cosmos saturn®, h/p/cosmos® sports & medical gmbh, Nusseldorf-Traunstein, Germany) using their own running shoes. To facilitate video analysis, cloth tapes were placed on the anterior and posterior talus, calcaneus, and mid-gastrocnemius on both limbs in each participant (Figure 2). The study protocol commenced with a 5-min warm-up session (Riley et al., 2018) where participants were allowed to transition from walking to running. Immediately after, the participants were directed to incrementally increase their running speed to a self-selected comfortable pace (9.4 ± 1.1 km/h) which they had to maintain for approximately 10 min. Verbal confirmation of their comfortable running speed was obtained prior to the start of the video recording. Participants were also instructed to maintain their running position at a consistent area of the treadmill. For recording the participants’ running, a single video camera (Casio Exilim EX-100) was utilized, and the camera was set at different spots for video recording from the front and back view. The video recordings were captured sequentially, with the front view being recorded first, followed by the back view.

**FIGURE 3:**
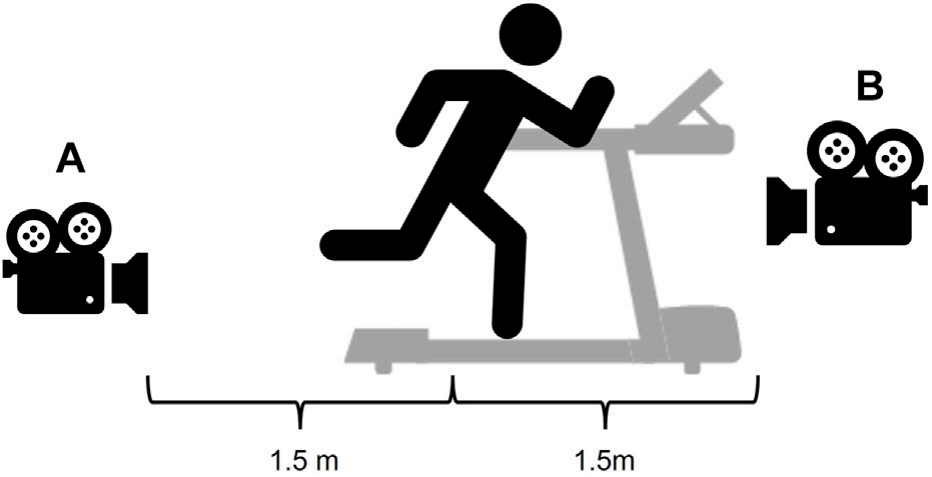
Illustration of video camera placements for recording in the **(A)** back camera view and **(B)** front camera view.

The video camera was placed 1.5 m away, perpendicular to the participants’ coronal plane (Figure 3). The camera height was set at the hip (front view) and ankle (back view) levels respectively for each participant. Each camera view was recorded for approximately 30 s at 120 Hz while participants were running at their self-selected speed during the 10-min window. Participants continued running while researchers move the camera position. Multiple recordings were taken in each camera position, and the video in which participants displayed minimal movement (moving sideways, etc.) was selected by the researchers for analysis.

**FIGURE 4:**
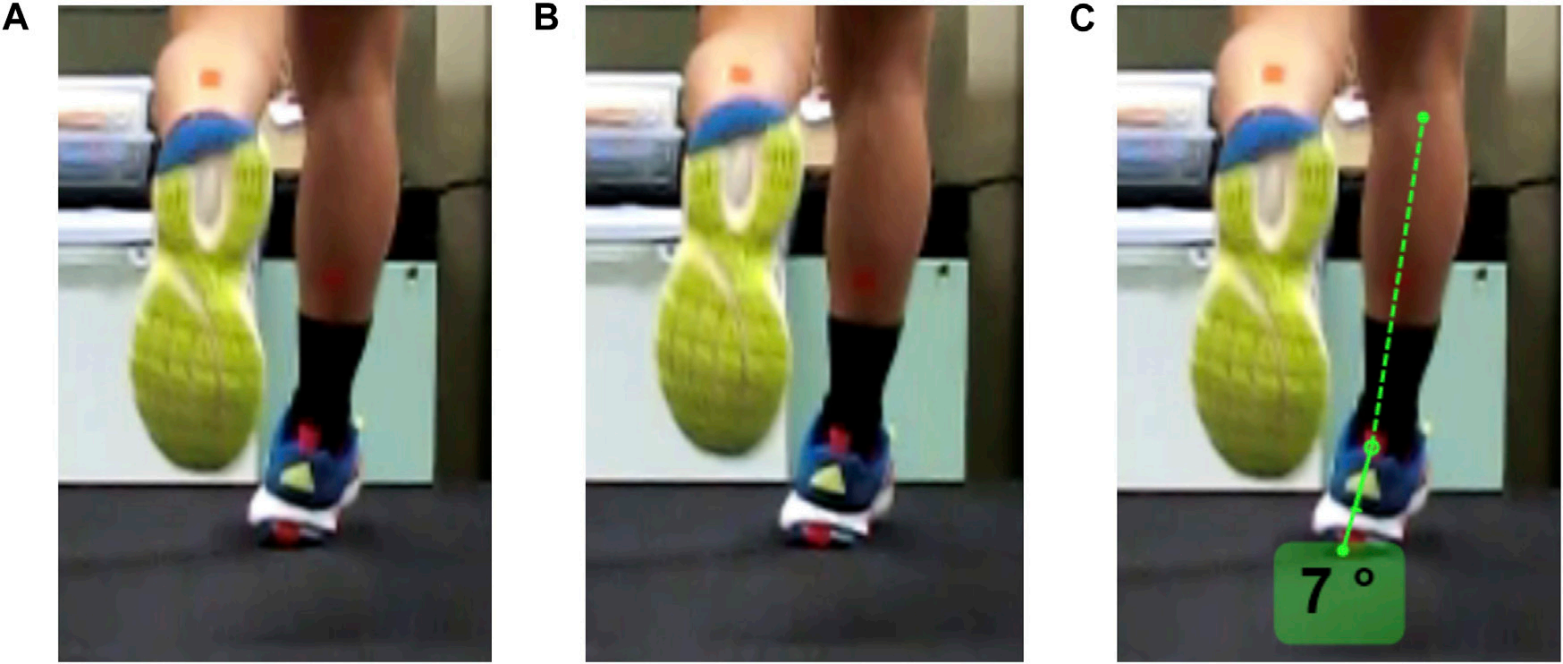
Frame-by-frame shots from the back view. **(A)** 1 frame before the initial foot strike, **(B)** frame of initial foot strike as heel contact is just made with the treadmill, and **(C)** angle measurements are derived from the line of posterior talus to the middle of gastrocnemius and calcaneus. Positive values indicate inversion while negative values indicate eversion.

**FIGURE 5:**
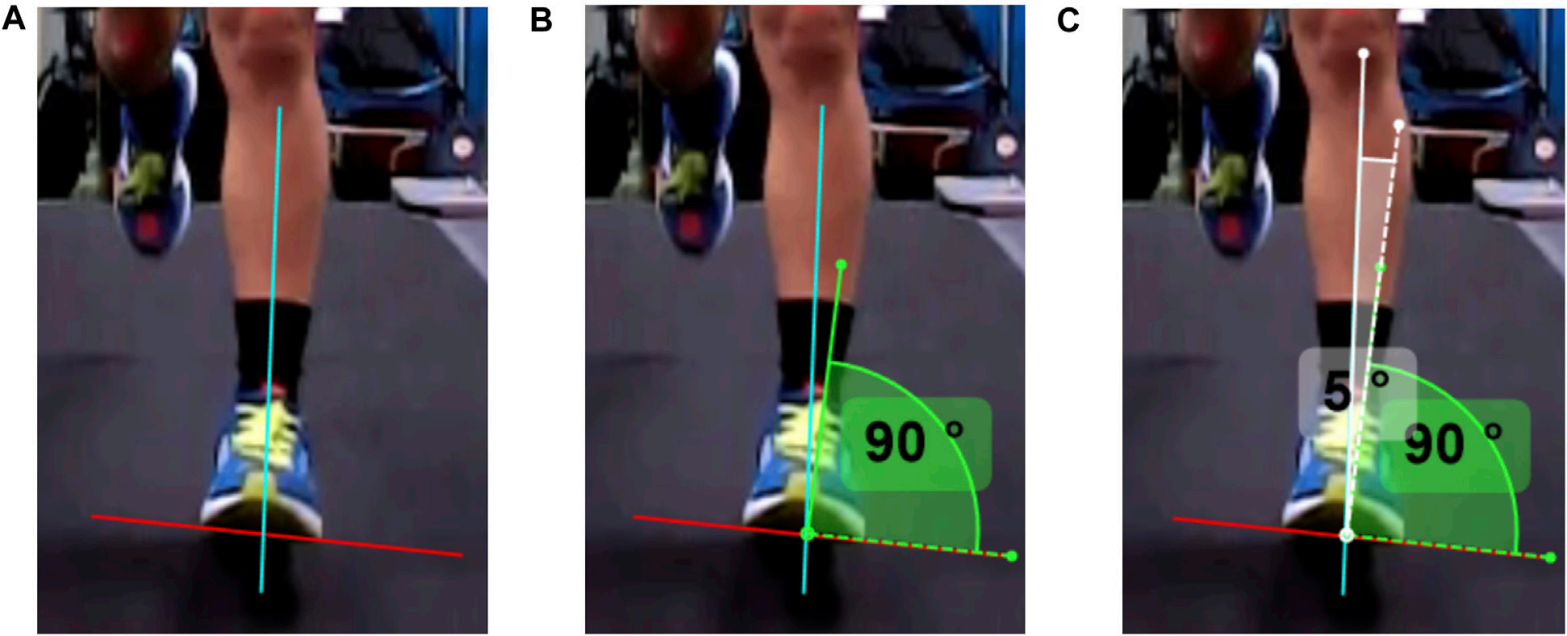
The sequence of steps taken to measure foot inversion at the initial foot strike from the front camera view. **(A)** In step 1, a parallel line (red) to the forefoot is drawn and a line (blue) from the base of the foot to the anterior talus. **(B)** In step 2, a right-angled (90°) measurement is placed at the intersecting point towards the lateral aspect of the foot. **(C)** In step 3, the angle is measured between the blue line to the relative right-angled measurement. Positive values indicate inversion while negative values indicate eversion.

Souza (2016) provided guidelines that were followed by researchers to standardize the identification of the initial foot strike frame. After identifying the frame of initial foot strike, the researchers used the following procedures to derive angle measurements for each camera view. In the back camera view, the inversion angle was calculated between the line extending from the posterior talus to the midpoint of the gastrocnemius and calcaneus (Figure 4). In the front camera view, the inversion angle was calculated by measuring the angle formed between the line from the base of the foot to the anterior talus and the perpendicular angle from the base of the foot (Figure 5). Positive values indicate inversion, while negative values indicated eversion in both measurement methods. Figures 4, 5 outline the steps taken in detail for angle measurements in the front and back camera views, respectively. Subsequently, 18 consecutive gait cycles of each foot from each camera view were analyzed in Kinovea (Version 0.8.27). The mean values across the 18 gait cycles were used for analysis. The reason for selecting 18 gait cycles was based on previous research indicating that a minimum number of steps, ranging between 7 and 25 gait cycles, were necessary to attain stable kinematic mean values (Dingenen et al., 2018a; Oliveira & Pirscoveanu, 2021).

### 2.3 Statistical analysis

To address the aims of the study, two types of analyses were conducted. Preliminary testing was first conducted to screen for normality and homogeneity of variance. As the assumption of normality was violated in the back view data according to the results of Shapiro-Wilk test, all data were log-transformed prior to statistical analysis. A 2 × 2 (Camera × Foot) Analysis of Variance (ANOVA) with repeated measures was performed to compare the measured angles from 2 camera views for each foot. As 2 × 2 ANOVA revealed no significant differences between the left and right feet, data from both feet were combined when performing the Pearson’s correlation test. Thereafter, linear regression was performed to derive a single equation for the conversion of the front-view angle to the typical back-view angle. The Wilcoxon signed-rank test was conducted to compare the equation-derived measurements and the actual measurement obtained from the back-view perspective for both the left and right foot. Effect size (r) was interpreted as small (0.1 ≤ r < 0.3), medium (0.3 ≤ r < 0.5), or large (0.5 ≤ r ≤ 1.0) (Cohen, 1988). Statistical significance was set at *p* < 0.05 level and the statistical analyses were performed using SPSS (Version 28; SPSS Inc., Chicago, IL, United States) and R (Version 4.1.1). As this study reported discrete values instead of continuous time-series kinematic data, no filtering was performed.

## 3 Results

**FIGURE 6:**
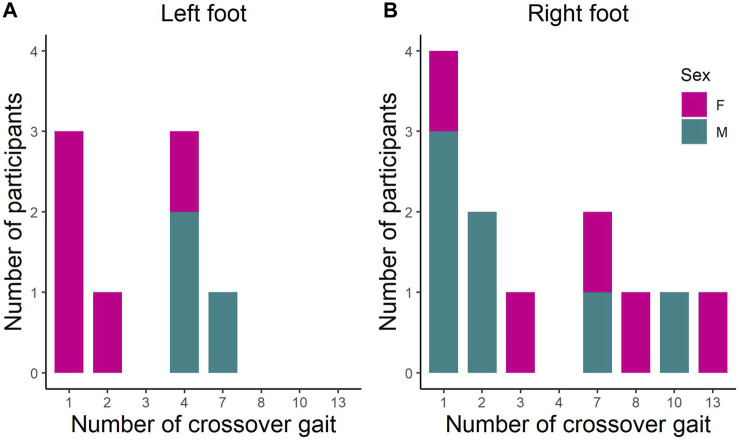
The number of crossover gait across 18 gait cycles in the **(A)** left foot and **(B)** right foot in male (M) and female (F) recreational runners.

The study observed that 13 out of 44 participants (29.5%) displayed at least one crossover gait within the 18 consecutive gait cycles in one foot (Figure 6). Seven participants (15.9%) had crossover gait in both feet while 8 (18.1%) and 12 participants (27.3%) showed crossover gait on the left and right foot, respectively.

Data are expressed as mean (95% confidence intervals). According to the results of the two-way ANOVA test, there was no significant main effect of Foot [F(1, 43) = 1.773, *p* = .190, η_p_^2^ = 0.040]. The mean angles were similar between the left and right foot in the front [left 10.9° (9.9°, 12.0°), right 11.8° (10.6°, 13.1°)] and back camera views [left 6.1° (4.6°, 7.6°), right 6.8° (5.2°, 8.4°)]. A significant main effect of Camera view was revealed [F(1, 43) = 62.542, *p* < .001, η_p_^2^ = 0.593], with front camera view measurements [11.4° (10.4°, 12.3°)] being greater than the back camera view measurements [6.5° (5.0°, 7.9°)] by 4.9° [3.5°, 6.3°]. No significant interaction effect was found between the Camera view and Foot [F(1, 43) = 0.230, *p* = 0.634, η_p_^2^ = 0.005].

**FIGURE 7:**
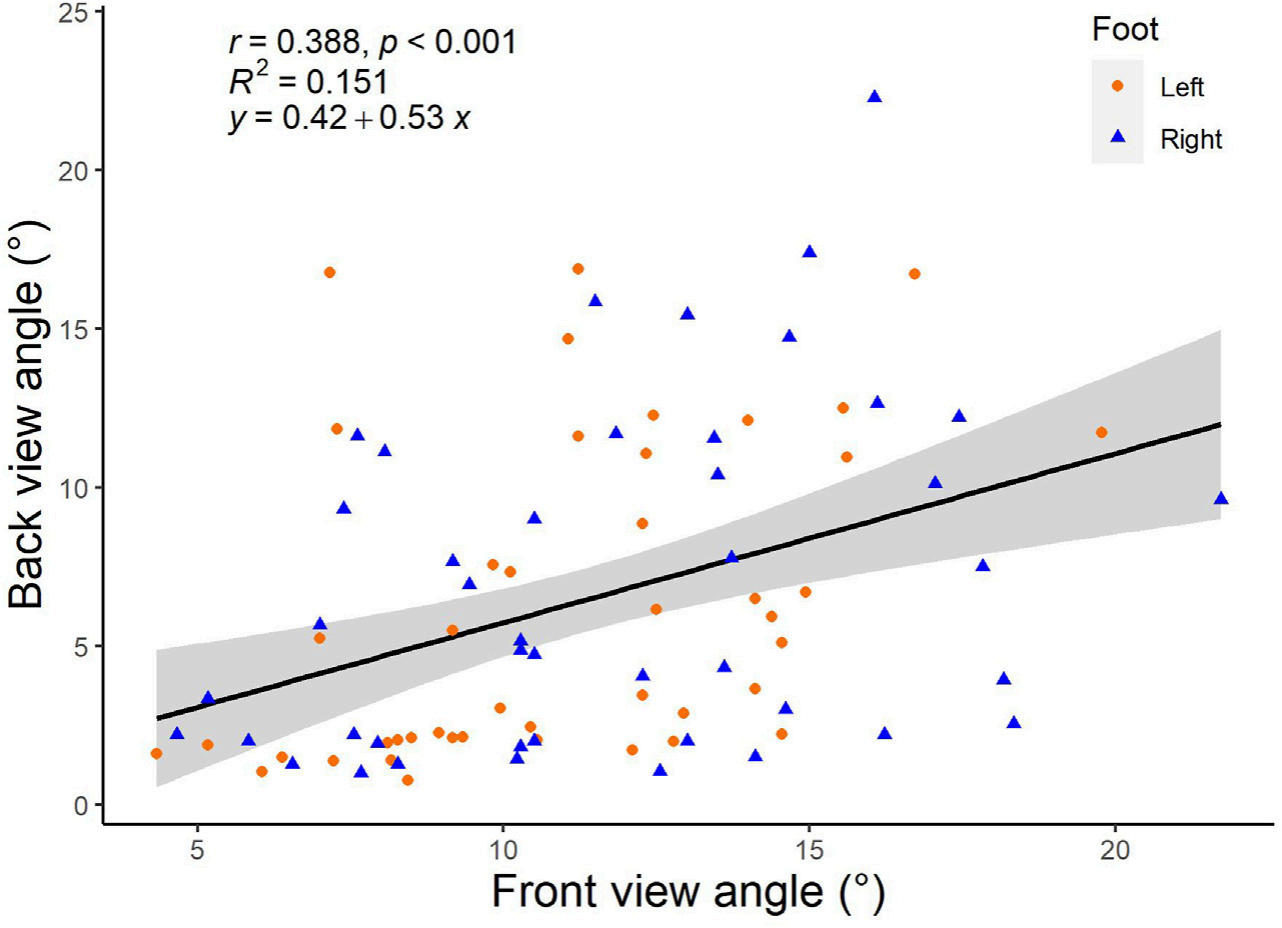
Correlation and regression analyses of the front-view and back-view foot inversion angles.

Pearson’s correlation test revealed a significant positive medium correlation (Figure 7) between the front and back camera view angles (*r* = 0.388, *p* < .001). Linear regression analysis also showed that front camera view measurement accounts for a significant amount of variance for measurements in the back camera view [F (1, 86) = 15.249, *p* < .001, *R* ^2^ = 0.151]. The regression coefficient (*β* = 0.53) allowed measurements from the back camera view to be predicted using the regression equation: y = 0.42 + 0.53 x , where y and x were the foot inversion angle measured from the back and front camera views, respectively. The mean angles from the equation-derived measurements are 6.2° (5.7°, 6.7°) for the left and 6.7° (6.1°, 7.3°) for the right foot. Subsequently, Wilcoxon signed-rank test revealed no significant difference between the means of equation-derived measurements and actual measurements for both left (Z = −0.805, *p* = 0.421) and right (Z = −0.140, *p* = 0.889) feet.

## 4 Discussion

This study aimed to investigate an alternative approach to measuring foot inversion angle from the frontal view, as opposed to the conventional back camera view measurement (Cornwall and McPoil, 1995). It was initially hypothesized that the front and back view measurements would be comparable. However, the results from this study suggest otherwise as front camera view measurements were significantly greater than back camera view measurements. Therefore, to facilitate comparisons with studies that commonly measure from the back view, an equation was provided to convert the front view measurements to the back view measurement. This conversion enables clinicians and practitioners to effectively utilize the back view measurements for comparative purposes.

### 4.1 Crossover gait

To provide a gauge on the frequency of crossover gait problems potentially encountered by clinicians and practitioners, the number of crossover gait was reported in this study. Approximately 30% of the participants in the present study displayed a crossover gait, resulting in an obstructed view of their foot when recording from the dorsal plane. This prevents practitioners from accurately measuring the foot inversion angle at the point of the initial foot strike when using the conventional technique. The frequent occurrence of crossover gait reaffirms that the traditional back-view measurement is insufficient and hence there is a need to look for alternative measurements, such as the front-view measurement proposed in our study.

### 4.2 Foot inversion angle

This study found that the mean front and back camera view measurements were 11.4° (10.4°, 12.3°) and 6.5° (5.0°, 7.9°), respectively. The traditional back camera view measurements were comparable with those obtained by McCrory et al. (1999), who also used a 2D video analysis approach and found that healthy runners displayed rearfoot eversion angles of −7.0° ± 0.7°. These results were similar to Grau et al. (2008), where a 3D motion analysis involving healthy runners revealed foot eversion angles of 1.9° ± 2.6°. In the studies mentioned above, the negative values denote an inversion angle and were measured from the back camera view. This emphasizes the caution that should be taken in using the values interchangeably. In addition, it also highlights the need for a standard conversion equation to be established, enabling front-view foot measurements to be translated and used in clinical diagnosis.

There are a few possible reasons for the differences in foot inversion angle measurements between the front and back camera views. For simplicity, this study treated the foot as a single rigid body. However, it is understood that the foot is a complex structure in which the forefoot and rearfoot may behave independently (Davis, 2004). This implies that the forefoot may have inverted without affecting the rearfoot. Therefore, the measurements could have differed significantly due to the independent foot structures.

No significant differences were revealed between the left and right foot inversion angles, suggesting that participants utilized comparable foot-landing techniques for both feet. This study did not examine individual foot strike patterns. However, previous research has shown that these techniques can impact rearfoot and forefoot kinematics. For example, heel-strikers are more likely to display greater inversion angles at touchdown compared with forefoot strikers (Pohl & Buckley, 2008). As such, this may have contributed to the discrepancies in measurements between the two camera views.

### 4.3 Regression analysis

The present study performed a regression analysis to obtain an equation that allows one to easily convert the front-view foot inversion angles to the traditional back-view rearfoot inversion angles. This regression equation can assist practitioners and clinicians in the event of an obstruction of the back camera view. For example, if a runner’s foot inversion angle is measured to be 10° from the front view, clinicians can calculate the supposedly back view angle to be approximately 5.7° (0.42 + 0.53 × 10°) and thereafter perform the necessary diagnosis for the runner. Additionally, the comparison between equation-derived measurements and actual measurements revealed no statistically significant difference. This confirms the possibility of using the equation to convert front view measurements to back view measurements despite having a low *R* ^2^ value. Future research could define reference norms and cut-off values based on front-view camera measurements of the foot inversion angle. This novel strategy might potentially be applied to all runners, regardless of the presence of crossover gait.

### 4.4 Limitations

This study used only one camera in each trial, positioned at different heights for the front and back views. The lack of synchronization between camera recordings may have compromised the accuracy of the results. The use of a single camera was done intentionally to simulate clinical settings where one camera view is often used to measure multiple kinematic variables, possibly due to the increased efficiency and limitations on manpower and equipment. For instance, the front camera view at the hip level could also measure knee adduction and contralateral hip drop (Dingenen et al., 2018a). Although this single-camera approach resulted in different gait cycles being analyzed and compared, it reflects the practical constraints encountered in clinical settings.

In addition, this study only measured 18 steps for each leg with varied gait patterns (e.g., crossover steps). Human locomotion such as running can present considerable variability between strides due to changes in motor control function (Stergiou et al., 2006). Previous studies that have attempted to identify the number of strides required for kinematic variables to reach a stable mean by performing a sequential estimation technique showed varying results. Dingenen et al. (2018a) reported a minimum of 7 steps while Oliveira and Pirscoveanu (2021) recommended a minimum of 25 steps were necessary to attain a stable kinematic mean. Therefore, although we have chosen a value within the higher range, it remains possible that the foot inversion angle measurements in this study did not reach a stable mean.

Another limitation is the use of a visual-based approach to identify the initial foot strike of running. This approach may be less accurate when compared to using an instrumented treadmill that provides ground reaction forces for quantitative identification (e.g., threshold of 20 N). From video recordings sampled at 60 Hz, Ghoussayni et al. (2004) found that the error in detecting the initial foot strike was less than 2 frames (0.033 s) when comparing to using a force platform. This present study used a high-speed video camera with 120 Hz recording frequency and hence a higher accuracy in initial foot strike identification can be expected.

## 5 Conclusion

This study explored the feasibility of measuring foot inversion angle at the initial foot strike of running from the front camera view. This method is particularly useful in crossover gait situations where the rearfoot is blocked in the back camera view. The findings indicate that the foot inversion angle at the initial foot strike is greater when measured from the front camera view than the back camera view. Researchers, practitioners, and clinicians are therefore cautioned against using measurements from different camera views interchangeably. A regression equation was also established to help clinicians translate the foot inversion angle measured from the front camera view to the standard rearfoot inversion angle measured from the back camera view. This will ensure accurate clinical diagnosis, using normative values that are currently derived from the back camera view.

## Data Availability

The raw data supporting the conclusion of this article will be made available by the authors, without undue reservation.
